# Cross-cultural adaptation and psychometric validation of the Dutch version of the Core Outcome Measures Index for the back (COMI -back) in patients undergoing surgery for degenerative disease of the lumbar spine

**DOI:** 10.1016/j.bas.2021.100004

**Published:** 2021-08-18

**Authors:** Pravesh S. Gadjradj, Mehrman Chalaki, Maurits W. van Tulder, Biswadjiet S. Harhangi

**Affiliations:** a Department of Neurosurgery, Park MC, Rotterdam, the Netherlands; b Department of Neurological Surgery, Weill Cornell Brain and Spine Center, New York-Presbyterian Hospital, New York, NY, USA; c Department of Neurosurgery, Leiden University Medical Center, Leiden, the Netherlands; d Department of Human Movement Sciences, Faculty of Behavioral and Movement Sciences, Amsterdam Movement Sciences Research Institute, Vrije Universiteit Amsterdam, the Netherlands; e Department of Neurosurgery, Erasmus MC University Medical Center Rotterdam, Rotterdam, the Netherlands

**Keywords:** Core outcomes measurement index, Low back pain, Patient reported outcome measure, Dutch, Validation, PROM, Patient-reported outcome measure, COMI, Core Outcomes Measures Index, RMDQ, Roland-Morris Disability Questionnaire, EQ5D, EuroQol-5 dimensions, WHOQoL-BREF, World Health Organization Quality of Life-BREF questionnaire, ICC, Intraclass correlation coefficient, SEM, Standard errors of measurement agreement, MDC, Minimum detectable change, SRM, Standardized response mean, ROC, Receiver-operating characteristic

## Abstract

**Introduction:**

Patient-reported outcome measures (PROMs) are the preferred outcomes measured in patients with lumbar spinal degenerative diseases. As PROMs can be lengthy and therefore pose a burden to patients and researchers, short and standardized PROMs are needed, such as the Core Outcome Measures Index (COMI).

**Research question:**

Is the Dutch version of the COMI-back a reliable and responsive PROM to measure outcomes in lumbar degenerative spinal surgery?

**Methods:**

After translating and cross-cultural adapting the COMI-back into Dutch, patients who were on the waiting-list for lumbar decompression surgery in a secondary referral center, were enrolled in the validation study. Patients completed a baseline booklet consisting of the COMI-back, likert scales measuring back and leg pain, the Roland-Morris Disability questionnaire, the EuroQoL-5 dimensions and the WHO-Quality-of-Life-BREF questionnaire to test construct validity. Within 2 weeks and before undergoing surgery, patients completed the COMI-back again, to measure test-retest stability. Three months after surgery, a global treatment outcome (GTO) question and the COMI-back were completed to test responsiveness.

**Results:**

The COMI-back was successfully translated and adapted into Dutch. One-hundred-thirty-five patients completed the baseline booklet, 93 the test-retest questionnaire and 102 the responsiveness questionnaire. The COMI-summary score and four of five COMI-domains, showed good to very good correlation to the reference questionnaires (ρ ​> ​0.41). The COMI-back showed a good test-retest stability with an intraclass correlation coefficient of 0.81 for the summary score. Furthermore, the standard error of agreement was 0.65 and the minimal detectable change was 1.8 points. The ROC-curve showed an area under the curve of 0.89 (95% CI 0.82 to 0.95).

**Conclusion:**

The Dutch version of the COMI-back has satisfactory psychometric properties and is a reliable and responsive patient-reported outcome measure in patients undergoing surgery for lumbar degenerative disease.

## Highlights

• The Core Outcome Measures Index for the back had yet to be validated among Dutch-speaking patients in the Netherlands.
• The COMI-back was validated among 135 patients who were candidate for lumbar spinal surgery. Construct validity, test-retest stability and responsiveness were tested.
• The COMI-summary score showed good to very good correlation to the reference questionnaires and a good test-retest stability. Furthermore, ROC-curve showed an area under the curve of 0.89 (95% CI 0.82 to 0.95).
• The Dutch version of the COMI-back has satisfactory psychometric properties and is a reliable and responsive PROM in patients undergoing surgery for lumbar degenerative disease.

## 1 Introduction

In degenerative spinal surgery, patient-reported outcome measures (PROMs) are the outcome measure of choice when outcomes of treatments are measured or compared between patients or patient groups (Deyo et al., 1998). Throughout the years multiple PROMs have been validated and are used in studies involving patients undergoing lumbar spine surgery (Ostelo et al., 2004; Trompenaars et al., 2005). These PROMs can cover one or more outcome domains such as pain or quality of life (Chiarotto et al., 2015). Because large studies are usually interested in multiple domains, patients are subsequently subjected to multiple PROMs which can be burdensome (Chiarotto et al., 2015). The Core Outcome Measures Index is a PROM measuring five domains using only seven questions (Mannion et al., 2009). These domains are (1) pain; (2) function; (3) symptom-specific well-being; (4) quality of life and (5) disability.

The COMI-back has been cross-culturally adapted and validated in several languages and among different patient groups and has become the PROM of choice for the The Spine Tango, the Eurospine Registry (Abdeldaiem et al., 2020; Damasceno et al., 2012; Klemencsics et al., 2016; Komesh et al., 2020; Mannion et al., 2016; Nagata et al., 2020; Qiao et al., 2013). A validation among Dutch patients or patients who can speak or read Dutch in the Netherlands, undergoing lumbar spine surgery, however, has not been conducted yet. Therefore, the purpose of the current study was to translate, cross-culturally adapt and validate the COMI-back in Dutch among patients undergoing surgery for lumbar degenerative spinal disease.

## 2 Methods

### 2.1 COMI-back

The COMI-back is a patient-reported outcome measure, that was intended to measure seven domains through seven questions. These domains are (1) low back pain; (2) leg pain; (3) function; (4) symptom-specific well-being; (5) quality of life; (6) social disability and (7) work disability. Low back pain and leg pain are measured on a numeric rating scale ranging from 0 to 10 with 10 indicating the “worst pain imaginable”. The other 5 domains are scored on a scale ranging from 0 to 5 with 5 indicating the “worst status”. These scores can be recalculated to scores ranging from 0 to 10 points. All seven domains can be used to calculate an overall COMI-summary score by selecting the highest pain score between low back pain and leg pain (worst pain score) and taking the average from work and disability scores (disability score). Then the COMI-summary score is calculated as the average of the worst pain score, the disability score, function, symptom-specific well-being and quality of life, and consequently ranges from 0 (best clinical status) to 10 (worst clinical status).

### 2.2 Translation process and cross-cultural adaptation

The English version of the COMI-back was translated to a Dutch version using established guidelines (Beaton et al., 2000). In brief, two native Dutch speakers (one involved in the field of spine surgery and one translator as layman) translated the English version to a Dutch version, independently. Afterwards, both translations were compared together with the original English version, which resulted in a first Dutch version. Subsequently, two native English speakers (one colleague and one layman) performed the back translation, independently. Both back translations were compared with the original English version.

All discrepancies were discussed during a research meeting, consisting of a clinician fluent in both languages and two bilingual researchers. All discrepancies were resolved in a group discussion and a pre-final Dutch version of the COMI-back was created. After institutional review board approval, this version was pilot tested among a subset of patients on a waiting list for lumbar decompression surgery. The pilot-testing did not result in any relevant interpretation issues and the pre-final version was finalized.

### 2.3 Patients

To further test the psychometric properties of the Dutch version of the COMI-back, the questionnaire was further validated among patients enrolled from two secondary referral centers for low complex spinal surgery. After their consultation with the neurosurgeon and after an indication for surgery was determined, patients were asked to participate in the current study. Patients were included if they were (1) at least 18 years of age; (2) a candidate for lumbar decompression surgery due to lumbar spinal stenosis and/or lumbar disk herniation. Exclusion criteria were (1) pregnancy; (2) infections or malignancy; (3) neurodegenerative disease; (4) lack of cooperation; (5) unable to understand Dutch and (6) serious psychopathology (to the discretion of the neurosurgeon).

### 2.4 Study procedures

After acquiring written informed consent, patients were requested to fill in a baseline booklet consisting of multiple PROMs. Within two weeks, and before undergoing surgery, patients were requested to return the retest questionnaire. Finally, three months after surgery, patients were asked to return the responsiveness questionnaire. All questionnaires were filled in by the patients without any assistance.

The baseline booklet consisted of (1) questions regarding patients' demographics; (2) the Dutch version of the COMI-back; (3) two five-point likert-scales measuring back and leg pain; (4) the Roland-Morris Disability Questionnaire; (5) the EuroQol-5 dimension questionnaire; and (6) the World Health Organization Quality of Life-BREF questionnaire. The Roland-Morris Disability Questionnaire (RMDQ) is a 24-question PROM measuring functional disability due to low back pain from 0 (no disability) to 24 (maximal disability) (Ostelo et al., 2004). The EuroQol-5 dimensions questionnaire (EQ5D) is a PROM consisting of 5 multiple choice answers and a 0 to 100 VAS measuring quality of life (MV et al., 2016). The World Health Organization Quality of Life-BREF questionnaire (WHOQoL-BREF) is a 26-question PROM covering four domains. The crude scores on the WHOQoL-BREF can be transformed to a 0 to 100 (best quality of life) summary score (Trompenaars et al., 2005).

The retest and responsiveness questionnaires contained the COMI-back and a global treatment outcome question (GTO) that is measuring self-reported recovery on a 7-point likert scale.

### 2.5 Analysis

All analyses were conducted using SPSS (version 24) with a p-value <0.05 indicating statistical significance. Missing data were handled as customary for each specific PROM. When the COMI-summary score is calculated, no missing answers were allowed for the analysis (Ostelo et al., 2004; Trompenaars et al., 2005; MV et al., 2016). To assess the psychometric properties of the COMI-back, (1) floor/ceiling effects; (2) construct validity; (3) test-retest stability and (4) responsiveness were measured (Trompenaars et al., 2005).

Floor/ceiling effects depict the percentage of patients which have the worst (floor) and best (ceiling) score on the PROM and do not reflect any meaningful deterioration or improvement in clinical status on the PROM. Descriptive statistics were used to measure floor/ceiling effects, and floor/ceiling effects were considered to be present if they were >15% and to be detrimental if they were >75%.

Construct validity involves the correlation between the new instrument, the COMI-back, and other validated PROMs. The Pearson's correlation coefficient (ρ) was used to evaluate the construct validity. A coefficient was considered to depict an excellent correlation with a ρ ​≥ ​0.81, very good correlation with a 0.61 ​≤ ​ρ ​< ​0.81, a good correlation with a 0.41 ​≤ ​ρ ​< ​0.61, a poor correlation with a 0.21 ​≤ ​ρ ​< ​0.40 and no correlation with a ρ ​< ​0.21 (Cohen, 1988; Steiner and Norman, 1995). It was hypothesized that all domains, except for the domain symptom-specific well-being, had a good correlation (ρ ​≥ ​0.41) for their reference questionnaires (see Table 3). Based on the literature, symptom-specific well-being was expected to have a fair correlation with the RMDQ and WHOQoL-BREF (physical function) (Nagata et al., 2020; Storheim et al., 2012; Genevay et al., 2012).

To assess reproducibility, test-retest stability was calculated. Reproducible tests should lead to similar test results with repeated testing when no change in clinical status occurs. To calculate the test-retest stability, only patients that indicated “no change in symptoms” on the GTO were included for this analysis. In these patients, the intraclass correlation coefficient (ICC) was calculated with corresponding 95% confidence interval for all COMI-domains and the COMI-summary score. An ICC >0.7 was considered to indicate acceptable reproducibility in a study sample of minimal 50 study participants (Terwee et al., 2007). Furthermore, standard errors of measurement agreement (SEM) and minimum detectable change (MDC) were calculated.

Responsiveness is the ability of an instrument to detect change in a clinical condition when it occurs. Responsiveness was measured in three ways (1) comparing scores on the COMI-back at baseline and at three months after surgery, using t-tests; (2) calculating the standardized response mean (SRM); (3) construct a receiver-operating characteristic (ROC) curve to evaluate sensitivity and specificity anchored on the GTO. An area under the curve of at least 0.70 was considered to be acceptable and the optimal cut-off score was determined using the Youden-method (Terwee et al., 2007; Youden, 1950).

## 3 Results

**Fig. 1:**
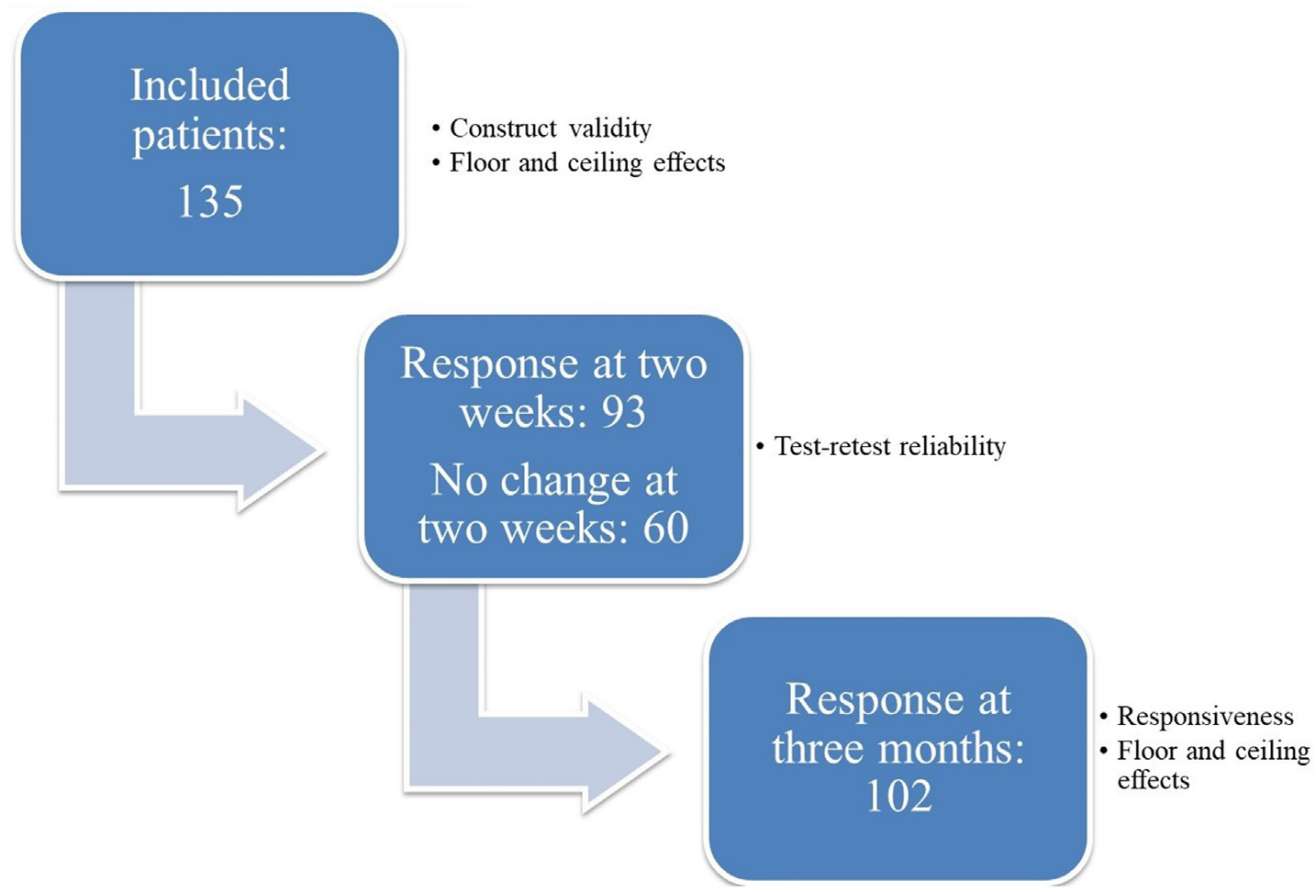
Flowchart of study procedures.

The COMI-back was successfully translated and is shown in the supplementary material. Items for discussion were mostly focused on cross-culture adaptations of items as “buttocks” and “pins and needles”. After performing the backward translations, no additional modifications were made to the COMI-back. Fig. 1 gives an overview of the study procedures. A total of 135 patients were included; 93 patients returned a re-test questionnaire and 102 patients returned the responsiveness questionnaire.

### 3.1 Demographics

Table 1 gives an overview of the baseline characteristics of the included patients. Patients had a mean age of 59.3 ​± ​15.4 years and 74.0% was married or in a relationship. Overall, 44.6% of the included patients had a paid job. The majority of the patients (45.4%) only had received lower education, while 24.6% had received middle level and 30.0% higher education. At baseline, 72.0% of the patients used pain medication.

**Table 1:** Baseline demographics of the included patients (N ​= ​135).

Characteristic	N (%)
Age (mean ​± ​SD)	59.3 ​± ​15.4

Marital status	131
*Married/in a relationship*	97 (74.0%)
*Single*	34 (26.0%)

Job status	130
*Paid job*	58 (44.6%)
*No (paid) job*	72 (55.4%)

Educational level	130
*Lower education*	59 (45.4%)
*Middle education*	32 (24.6%)
*Higher education*	39 (30.0%)

Use of the following:	
*Cigarettes/tobacco*	38 (28.8%)
*Antidepressants*	9 (6.8%)
*Muscle relaxants*	4 (3.0%)
*Pain medication*	95 (72.0%)

Patient-reported outcome measure (mean ​± ​SD)	
*Rolland-Morris Disability Questionnaire*	15.8 ​± ​4.9
*WHOQOL-BREF*	56.8 ​± ​8.7
*EQ5D*	0.723 ​± ​0.0751
*COMI-summary score*	7.5 ​± ​1.4

### 3.2 Acceptability and floor/ceiling effect

On average, patients completed the COMI-back in four and a half minutes. Table 2 gives an overview of the percentage missing data of the COMI-back at baseline and the floor/ceiling effects at baseline and at 3-months follow-up. Missing data ranged from 0 to 4.4% on the different COMI-domains. The domains low back pain, leg pain, social and functional disability, and the summary score had missing items.

**Table 2:** Floor and ceiling effects at baseline and at 3-months of follow-up.

	Min-max	Missing at baseline%	Baseline		3-months follow-up	
			Floor effects % (N)	Ceiling effects % (N)	Floor effects % (N)	Ceiling effects % (N)
COMI low back pain	0–10	2 (1.5%)	3 (2.3%)	3 (2.3%)	0	17 (16.2%)
COMI leg pain	0–10	2 (1.5%)	8 (6.0%)	0	1 (1.0%)	22 (21.0%)
COMI function	0–10	0	43 (31.9%)	4 (3.0%)	12 (11.4%)	11 (10.5%)
COMI symptom-specific well-being	0–10	0	110 (81.5%)	0	31 (29.5%)	12 (11.4%)
COMI quality of life	0–10	0	17 (12.6%)	1 (0.7%)	8 (7.6%)	5 (4.8%)
COMI social disability	0–10	1 (0.7%)	73 (54.5%)	3 (2.2%)	29 (27.6%)	26 (24.8%)
COMI work disability	0–10	2 (1.5%)	55 (41.4%)	19 (14.3%)	30 (29.7%)	39 (38.6%)
COMI summary score	0–10	6 (4.4%)	1 (0.8%)	0	0	1 (1.0%)

At baseline, the domains function (31.9%) and the social- (54.5%) and work (41.4%) disability showed high floor effects. Only the domain symptom-specific well-being (81.5%) showed high ceiling effects at baseline. At three months, the domains symptom-specific well-being (29.5%) and the domains social- (27.6%) and work (29.7%) disability had high floor effects. Furthermore, high ceiling effects were present at the domain of low back pain (16.2%), leg pain (21.0%) and social- (24.8%) and work (38.6%) disability at three months follow-up.

### 3.3 Construct validity

Table 3 gives an overview of the correlation tests of the COMI-back with the other questionnaires tested at baseline. The COMI domains low back pain and leg pain showed a very good correlation with the likert scales for low back pain (0.77) and leg pain (0.64). The COMI domain function showed a good correlation with the RMDQ (0.41) and WHOQoL-BREF Physical function (−0.47). The domain symptom-specific well-being showed a poor correlation with the RMDQ (0.27) and WHOQoL-BREF Physical function (−0.33), and no correlation with the WHOQoL total score (−0.18). The COMI domain quality of life showed good correlation with the EQ5D (−0.49) and EQ5D-VAS (−0.45), but poor correlation with the WHOQoL-BREF (−0.40). The COMI domains social- and work disability showed good correlations with the RMDQ (0.47 and 0.53, respectively) and with the WHOQoL-BREF (both −0.47). Finally, the overall COMI summary score showed a good correlation with the EQ5D (−0.51) and the WHOQoL-BREF (−0.47), and a very good correlation with the RMDQ (0.63) and WHOQoL-BREF Physical function (−0.65).

**Table 3:** Construct validity.

COMI-items	Reference questionnaire	Pearson ρ
Low back pain	Likert low back pain	0.77∗
Leg pain	Likert leg pain	0.64∗
Function	RMDQ	0.41∗
	WHOQoL-BREF Physical function	−0.47∗
Symptom-specific well-being	RMDQ	0.27∗
	WHOQoL-BREF Physical function	−0.33∗
	WHOQoL-BREF	−0.18
Quality of life	EQ5D	−0.49∗
	EQ5D-VAS	−0.45∗
	WHOQoL-BREF	−0.40∗
Social disability	RMDQ	0.47∗
	WHOQoL-BREF Physical function	−0.47∗
Work disability	RMDQ	0.53∗
	WHOQoL-BREF Physical function	−0.47∗
Summary score	RMDQ	0.63∗
	EQ5D	−0.51∗
	WHOQoL-BREF Physical function	−0.65∗
	WHOQoL-BREF	−0.47∗

∗p-value<0.001.

### 3.4 Test-retest reliability

Of the 93 patients that returned the test-retest questionnaire, 60 experienced no change in symptoms based on the GTO-question. Table 4 gives an overview of the results of the test-retest reliability analyses. Except for the domains function (0.53), symptom-specific well-being (0.51) and quality of life (0.51), all domains (0.75–0.88) and the summary score (0.81) showed good test-retest stability. The COMI summary score had a SEM of 0.65 and a MDC_95%_ of 1.8.

**Table 4:** Test-retest reliability.

COMI domains	Mean (±SD) 1st measurement	Mean (±SD) 2nd measurement	ICC (95% CI)	SEM	MDC 95%
Low back pain	5.7 ​± ​2.6	5.6 ​± ​2.5	0.75 (0.61–0.85)	1.30	3.6
Leg pain	7.5 ​± ​1.7	7.2 ​± ​1.8	0.88 (0.80–0.93)	0.59	1.6
Function	7.5 ​± ​2.5	7.5 ​± ​2.1	0.53 (0.32–0.69)	1.71	4.7
Symptom-specific well-being	9.6 ​± ​0.9	9.3 ​± ​1.2	0.51 (0.29–0.67)	0.63	1.7
Quality of life	6.3 ​± ​2.0	6.2 ​± ​2.0	0.51 (0.29–0.67)	1.40	3.9
Social disability	7.5 ​± ​3.0	7.2 ​± ​3.2	0.85 (0.76–0.91)	1.20	3.3
Work disability	6.3 ​± ​3.9	6.6 ​± ​3.9	0.88 (0.81–0.93)	1.35	3.7
Summary score	7.4 ​± ​1.5	7.2 ​± ​1.5	0.81 (0.67–0.89)	0.65	1.8

### 3.5 Responsiveness

**Fig. 2:**
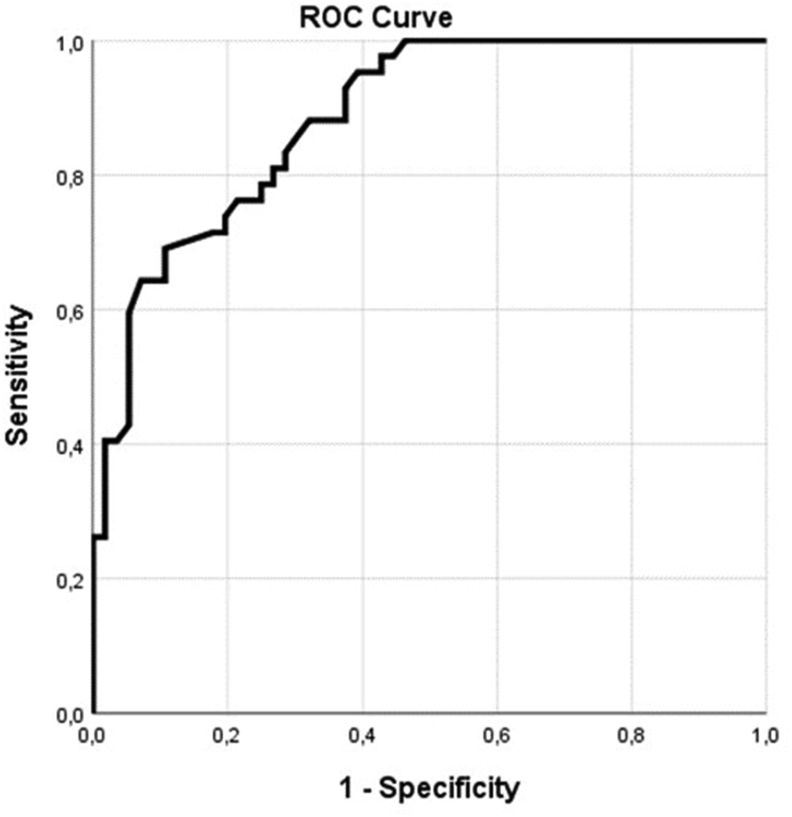
Receiver-operating characteristic curve.

Using the dichotomized likert-scale for recovery, 57.3% of the patients were fully recovered at three months follow-up. Patients that recovered had a mean COMI-summary score of 3.2 ​± ​2.0 compared to 6.6 ​± ​1.8 (mean difference −3.3, p ​< ​0.001). The SRM was 1.1 overall. The SRM for a good outcome was 2.0 while the SRM for a poor outcome was 0.65. Fig. 2 depicts the ROC-curve of the COMI-summary score anchored for recovery. The area under the curve was 0.89 with 95% CI (0.82–0.95). The optimal cut-off was 2.0 points and yielded a sensitivity of 69% and specificity of 89%.

## 4 Discussion

This study presents the cross-culturally adapted and validated Dutch version of the COMI-back. The COMI-back was validated among 135 patients who underwent surgery for degenerative lumbar spinal disease. The COMI-back had only a small percentage of missing answers and the COMI-summary score had negligible floor and ceiling effects. One domain had detrimental floor effects at baseline, while the COMI-summary score had no floor and ceiling effects. Furthermore, the COMI-summary score and all COMI domains, except for the domain symptom-specific well-being, showed good to very good correlation to the reference questionnaires. Test-retest stability was good for the summary score and responsiveness analysis showed a good discriminatory ability of the COMI-back between patients with a good and patients with a poor outcome.

Recently, a Dutch version of the COMI-back was cross-culturally adapted and validated among Dutch-speaking Belgian patients suffering from low back pain with or without leg pain (Van Lerbeirghe et al., 2018). It is unknown of these patients were candidates for surgery or conservative treatment. Both Dutch versions show many similarities, but also some cultural differences. For instance, words like “gelieve (please)”, “job (job)”, “omwille (because)”, are more commonly or only used in the Dutch that is spoken in Flanders, Belgium. The ‘Flemish Dutch’ validation show that the COMI-back is a valid and reliable patient-reported outcome measure in patients with at low back pain while the current ‘Holland Dutch’ validation confirms this among patients on a waiting list for lumbar decompression surgery. Furthermore, the currents study also demonstrated good responsiveness of the COMI-back.

In the current study, the domains function and disability showed high floor effects, while symptom-specific well-being showed detrimental floor effect at baseline. Although floor and ceiling effects are unfavorable for PROMs in general, these effects are not unknown to the COMI (Damasceno et al., 2012; Klemencsics et al., 2016; Storheim et al., 2012; Miekisiak et al., 2013). The unexpected high floor effect in the domain symptom-specific well-being, may also be partially explained by our study population, as invalidating pain or disability is part of the indication for surgery. Nevertheless, the COMI-summary score showed negligible floor and ceiling effects and therefore only a small impact is expected of the floor and ceiling effects of the individual domains.

Except for one domain, all COMI-domains and the summary score showed good to very good correlation with the index questionnaires. Both the (very) good and poor correlations shown in the current study are comparable to the correlations found in validation studies in other languages of the COMI-back (Abdeldaiem et al., 2020; Damasceno et al., 2012; Klemencsics et al., 2016; Storheim et al., 2012; Van Lerbeirghe et al., 2018; Miekisiak et al., 2013; Kim et al., 2018). Furthermore, also the test-retest reliability and MDC-scores calculated in the current study seem to be in line with the literature (Abdeldaiem et al., 2020; Damasceno et al., 2012; Klemencsics et al., 2016; Komesh et al., 2020).

The responsiveness of the COMI-back has only been assessed in a few other studies (Klemencsics et al., 2016; Mannion et al., 2005). In the validation study of the Hungarian version of the COMI-back, the responsiveness was tested in 159 patients, six months after they underwent surgery for the lumbar spine. In a German validation study, similarly, responsiveness’ was tested in a surgical population (Mannion et al., 2005). The ROC-curves of the Hungarian and German versions had an area under the curve of 0.84 and 0.82, respectively. In the current study an area under the curve of 0.89 was found, indicating comparable and good discriminatory abilities of the COMI-back.

Some limitations of the study methodology have to be acknowledged. The Dutch language may have some cultural differences between the Dutch-speaking countries the Netherlands and Belgium. Therefore, this version may not be as applicable in other Dutch speaking regions, such as Surinam and the Netherlands Antilles. In the current study, we tried to prevent the use of region-specific language. Another limitation may be the use of the Pearson ρ to test the correlation as it may be debatable that the use of the Spearman ρ might have been more appropriate. Post-hoc analyses using the Spearman ρ coefficient, showed only small differences in correlation compared to the Pearson ρ coefficient and did not change the conclusion. Strengths of this study include the adherence to guidelines for the adaptation of PROMs, the appropriate sample sizes to perform the psychometric testing, the inclusion of a GTO-question while measuring the test-retest reliability, the inclusion of testing the responsiveness and the inclusion of patients from multiple clinics in the Netherlands. Specific strengths of applying the COMI-back in practice includes the short time needed to complete the questionnaire and the low burden to patients and researchers.

The Dutch version of the COMI-back has satisfactory psychometric properties and is a reliable and responsive patient-reported outcome measure in patients undergoing surgery for lumbar degenerative disease. As the use of the COMI-back is easy to understand and takes only about four and a half minutes to complete, we recommend using it as a patient-reported outcome measure in studies and registries focused on degenerative lumbar spinal surgery.

## Declaration of competing interest

All authors (PG, MC, MvT and BSH) have no conflict of interest to disclose. ICMJE-forms of all authors are available upon request.
