# Supplementary material for: Cross-cultural adaptation and psychometric validation of the Dutch version of the Core Outcome Measures Index for the back (COMI -back) in patients undergoing surgery for degenerative disease of the lumbar spine
Source: Brain Spine. 2021 Aug 18;1:100004. doi: 10.1016/j.bas.2021.100004 (PMC9560685; doi:10.1016/j.bas.2021.100004)
Supplement: Multimedia component 1 [file mmc1.docx]

**Dutch translated version of the COMI-back**

**Rugproblemen** kunnen leiden tot rugpijn en/of pijn in de benen/ het zitvlak. Daarnaast kunnen er ook gevoelsstoornissen zoals tintelingen, een prikkend gevoel of een doof gevoel in deze gebieden ontstaan.

1 Van welk van de volgende problemen 🞏_1_ rugpijn

heeft u **het meeste** last? 🞏_2_ pijn in de benen/ het zitvlak

Kruis **één optie** aan. 🞏_3_ gevoelsstoornissen in de rug/de benen/ het zitvlak zoals een tintelend of doof gevoel, of een prikkend gevoel.

🞏_4_ geen van bovenstaande opties

2 Voor de volgende 2 vragen (vraag 2a en vraag 2b) zouden we u willen vragen de ernst van uw pijn aan te geven door een kruisje te zetten op de schaal van 0-10 (waarbij “0”= geen pijn, “10”=de ergste pijn die u zich kunt voorstellen).

Er zijn aparte vragen voor rugpijn en pijn in de benen/billen.


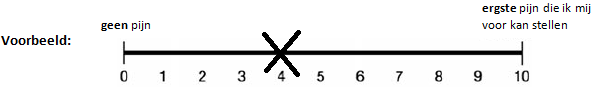


2a Hoe erg was uw **rugpijn** in de afgelopen week?


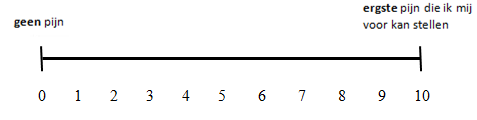


2b Hoe erg was de pijn in uw **benen (ischias) /zitvlak** de afgelopen week?


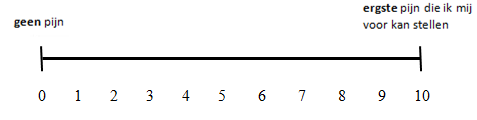


3 Gedurende de **afgelopen week**, in welke 🞏_1_ helemaal niet

mate stond uw rugprobleem uw **normale** 🞏_2_ een klein beetje

**activiteiten** (zowel werk buitenshuis 🞏_3_ matig

als in het huishouden) in de weg ? 🞏_4_ best wel

🞏_5_ uiterst

4 Als u **de rest van uw leven** zou moeten 🞏_1_ erg tevreden

leven **met de klachten die u nu heeft**, 🞏_2_ enigszins tevreden

hoe zou u zich daar dan bij voelen? 🞏_3_ noch tevreden noch ontevreden

🞏_4_ enigszins ontevreden

🞏_5_ erg ontevreden

5 Terugkijkend op de **afgelopen week**, 🞏_1_ erg goed

hoe zou u uw kwaliteit van leven beoordelen? 🞏_2_ goed

🞏_3_ gematigd

🞏_4_ slecht

🞏_5_ erg slecht

6 **Gedurende de afgelopen 4 weken**, hoeveel 🞏_1_ geen

dagen heeft u moeten minderen met **de** 🞏_2_ tussen de 1 en 7 dagen

**activiteiten die** **u doorgaans onderneemt** (uw werk, 🞏_3_ tussen de 8 en 14 dagen

het huishouden, school, ontspannende activiteiten) 🞏_4_ tussen de 15 en 21 dagen

vanwege uw rugprobleem? 🞏_5_ meer dan 21 dagen

7 **Gedurende de afgelopen 4 weken**, hoeveel 🞏_1_ geen

dagen heeft uw rugprobleem **u ervan** 🞏_2_ tussen de 1 en 7 dagen

**weerhouden** **om te gaan werken** (uw werk, school, 🞏_3_ tussen de 8 en 14 dagen

het huishouden)? 🞏_4_ tussen de 15 en 21 dagen

🞏_5_ meer dan 21 dagen
